# In Situ Enzymatic Polymerization of Ethylene Brassylate Mediated by Artificial Plant Cell Walls in Reactive Extrusion

**DOI:** 10.1021/acsapm.4c01568

**Published:** 2024-08-16

**Authors:** Luca Deiana, Angelica Avella, Abdolrahim A. Rafi, Rosica Mincheva, Julien De Winter, Giada Lo Re, Armando Córdova

**Affiliations:** † Department of Natural Sciences, Mid Sweden University, Holmgatan 10, Sundsvall 85179, Sweden; ‡ Department of Industrial and Materials Science, Chalmers University of Technology, Rännvägen 2a, Gothenburg 41258, Sweden; § Laboratory of Polymeric and Composite Materials, University of Mons (UMONS), 7000 Mons, Belgium; ∥ Organic Synthesis and Mass Spectrometry Laboratory (S2MOs), University of Mons (UMONS), 7000 Mons, Belgium

**Keywords:** poly(ethylene brassylate), artificial plant cell wall, macrocycles, ring-opening polymerization, reactive extrusion, solvent-free, ethylene brassylate, metal-free catalysis

## Abstract

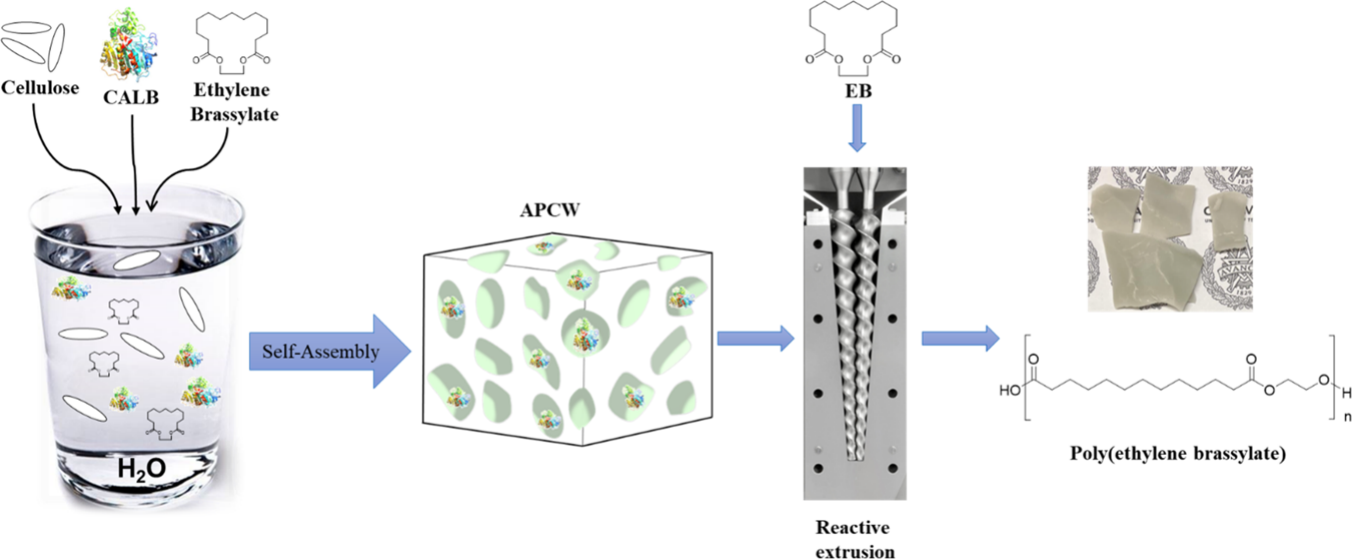

Herein, we describe a solvent-free bioinspired approach for the polymerization of ethylene brassylate. Artificial plant cell walls (APCWs) with an integrated enzyme were fabricated by self-assembly, using microcrystalline cellulose as the main structural component. The resulting APCW catalysts were tested in bulk reactions and reactive extrusion, leading to high monomer conversion and a molar mass of around 4 kDa. In addition, we discovered that APCW catalyzes the formation of large ethylene brassylate macrocycles. The enzymatic stability and efficiency of the APCW were investigated by recycling the catalyst both in bulk and reactive extrusion. The obtained poly(ethylene brassylate) was applied as a biobased and biodegradable hydrophobic paper coating.

## Introduction

The quest for alternative ecofriendly protocols for the synthesis of organic molecules is becoming one of the predominant aspects in the chemistry field. The transition from fossil-based raw materials to green and renewable sources has increased exponentially in the past few years.^1,2^ Cellulose is the most abundant macromolecule on earth.^3^ Its large availability, biocompatibility, biodegradability, and stability^2^ make this natural polymer a perfect candidate for heterogeneous scaffold for enzymatic biocatalysis.^4−6^ As observed and demonstrated by kinetic experiments, the enzymatic activity of biomolecules decreases in organic solvents and nonaqueous systems.^7,8^ To circumvent this intrinsic chemostructural problem, a plethora of solid supports have been screened as carriage to shield the enzyme from a nonfavorable environment.^9−12^ Many different materials such as silica,^13,14^ resins,^15^ synthetic polymers,^16^ carbon nanotubes,^17^ and graphene^18^ have been used for this scope. In parallel, various immobilization techniques such as adsorption,^19^ covalent binding,^20^ entrapment,^12^ encapsulation,^21^ cross-linking,^22^ and 3D printing^23^ were developed. One of the main issues of these strategies lies in the complexity of the protocols, implicating tedious extra synthetic steps and a huge waste of chemicals and solvents. All of these drawbacks, in most cases, lead to the confinement of the biocatalysts merely to lab-scale applications and theoretical studies. Thus, for ton-scale industrial processes innovative routes need to be explored, fulfilling the modern environmental regulations that have been recently promulgated.^24^ In the field of enzymatic polymerization, the choice of medium and reaction conditions is balanced by a fragile equilibrium between the desired polymer characteristics and the biocatalyst stability.^25,26^ However, most of the protocols involve harsh reaction conditions, such as high temperatures or low vacuum pressure, to remove the generated volatile byproducts, which may denature the enzyme during the process.^27−30^ Efforts were made to switch from highly toxic organic solvents to water, showing obvious major economic and environmental benefits.^31−33^ The drawback of using water, as with all other solvents, lies in the much larger ratio between reactor volume and product obtained. Also, the necessity of extra plants for solvent purification before disposal has a negative economic and environmental impact.

**Figure 1:**
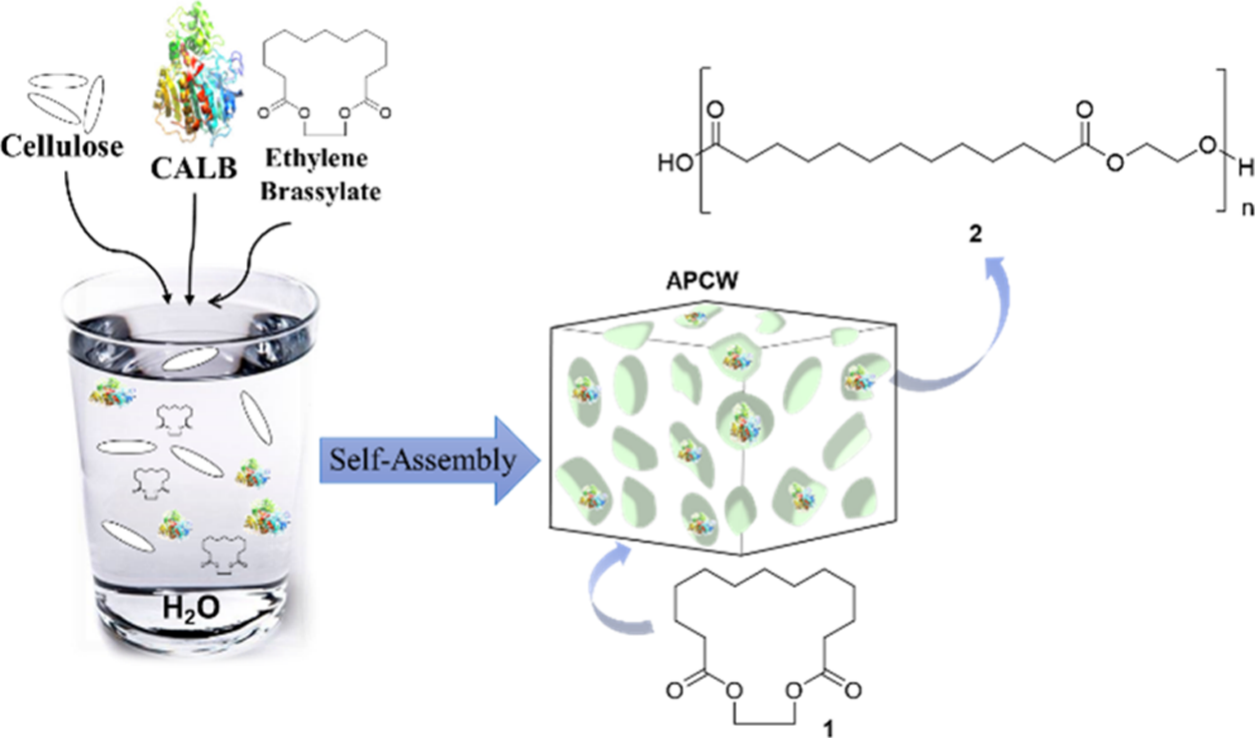
Simplified scheme of the self-assembly of components to create an APCW, which catalyzes the polymerization of ethylene brassylate.

In our group, we have a long experience concerning the valorization of biomass, the construction of functional cellulose-based materials,^34,35^ and heterogeneous catalysis.^36−38^ Recently, we disclosed a concept regarding the construction of polysaccharide-based heterogeneous enzyme/metal catalyst systems mimicking natural plant cell walls or arthropod exoskeletons for application in asymmetric catalysis.^39^ These sustainable heterogeneous catalysts were successful in catalyzing the dynamic kinetic resolution of racemic primary amines. Motivated by our previous success, we decided to further expand our concept and face challenges in polymer synthesis. Inspired by the natural tridimensional structural complexity of the primary plant cell wall, we envisioned a tailor-made and self-assembled artificial plant cell wall (APCW) able to promote the heterogeneous polymerization of ethylene brassylate under solvent-free conditions (Figure 1).

Ethylene brassylate (EB, **1**) is a commercially available renewable macrolactone derived from castor oil. Poly(ethylene brassylate) (PEB, **2**), a biodegradable aliphatic polyester, is synthesized by ring-opening polymerization of the macrolactone. Previous studies reported EB polymerization through enzymatic,^40^ organometallic,^41−47^ or organic^42,48−50^ catalysis. Compared to other catalysis routes, enzymatic polymerization is a more environmentally benign process, and it has the advantages of low toxicity, activity at low temperatures, and the enzymes can be naturally sourced.^51^

Reactive extrusion (REx) is a one-step processing technique that can enable polymerization without the need for solvents. The extrusion process improves the mixing and lowers the viscosity of the reaction compared to conventional bulk conditions; thus, it can promote the reaction kinetics. Spinella et al.^52^ demonstrated that REx is faster and leads to higher molecular weight than bulk or solution conditions for the lipase-catalyzed ring-opening polymerization of ω-pentadecalactone. Recently, we conducted organocatalytic EB polymerization using REx for the production of nanocomposites.^53^

Growing environmental concerns regarding the use of single-use plastics are pushing the use of paper-based materials due to their renewability and biodegradability. However, the porous paper structure and its hydrophilicity result in a poor moisture resistance. Numerous coating methods have been developed, mainly utilizing metals or fossil-based nondegradable polymers^54^ which hinder paper biodegradation. Their replacement with biobased and biodegradable coating would be advantageous.

The objective of this work is to design and test the activity of bioinspired artificial plant cell walls as catalysts for the polymerization of ethylene brassylate and to investigate how different reaction conditions (bulk vs REx) affect the polymerization. Herein, we disclose the successful use of APCWs as a catalyst for sustainable ring-opening polymerization of EB. In order to promote the transition from fossil-based feedstocks to circular renewable materials, the produced PEB was successfully tested as a biobased and biodegradable paper coating.

## Results and Discussion

APCW catalyst was self-assembled from a mixture of cellulose, enzyme, and surfactant (Figure 1, Table S1). The cellulose source chosen, as a structural component of the biocatalyst APCW, was microcrystalline cellulose (MCC) due to its intrinsic natural characteristics^55^ as well as the low price and easy attainability (MCC Avicel PH-101 can be obtained for 2.00 US$-3.50 US$/kilogram, 20 kg min order). As the enzyme/protein component, we chose *Candida antarctica* Lipase B (CALB) and for the surfactant Brij or EB.

Our investigation started with the screening of the influence of different APCW components on the outcome of the EB polymerization monitored by ^1^H NMR (Figures S1 and S2). The assignment of PEB signals was supported by a COSY NMR experiment (Figure S3). First, the effect of neat CALB on the synthesis of PEB was evaluated. Pure lyophilized CALB without modifications afforded only traces of the product after 72 h (entry 1, Table 1). Then, MCC was modified with CALB and phosphate buffer to prepare APCW1, leading to 80% conversion and a molar mass (*M*_w_) of 2160 g/mol after 5 h (entry 2, Table 1). Adding the surfactant Brij to the composition of APCW2 increased the rate of the reaction leading to 94% conversion and *M*_w_ of 2295 g/mol in 2 h (entry 3, Table 1). Removing phosphate buffer from the catalyst components and, instead, using distilled water as a freeze-drying medium for the self-assembly of APCWs permitted the reduction of the mass of the biocatalyst in correlation with the amount of pure CALB contained (entries 2 and 4, Table 1). APCW3 afforded 80% conversion in 4 h and *M*_w_ of 4860 g/mol (entry 5, Table 1). Adding Brij as the surfactant, in the absence of buffer, speeded up the reaction rate to 1 h and 93% conversion, but the *M*_w_ slightly decreased to 2970 g/mol (entry 6, Table 1). Aware of the chemical similarities between Brij and ethylene brassylate, we decided to switch from Brij surfactant to the polymer monomer in the self-assembly of APCW5. The polymerization afforded 91% conversion and the best value for *M*_w_ in just 3 h (entry 7, Table 1). In comparison, Müller et al.^40^ carried out enzymatic polymerization of ethylene brassylate in bulk using commercially available CALB as a catalyst immobilized on a fossil-based polymer support (Novozyme 435). In this case, CALB catalyzed the ROP of EB in 91% conversion to reach a molecular weight of 3500 g/mol after a reaction time of 6 h. Thus, sustainable APCW systems are more efficient. The APCW5 catalyst was recovered from the reaction shown in entry 7 and reused to catalyze a new polymerization reaction (entry 8, Table 1). As shown in the screening in Table 1, APCW5 is still active, leading to an increase in the polymer size to *M*_w_ of 6750 g/mol. The longer reaction time is probably due to the viscosity of the system affecting the stirring rate. Additional recycling further slowed down the polymerization. Based on previous studies that α-hydroxy acids (e.g., tartaric acid, lactic acid) can catalyze the direct ring-opening polymerization of lactones,^56^ we screened their efficiency on ethylene brassylate. As shown in the table, only traces of products were formed after 91 h (entry 9, Table 1).

**Table 1:**
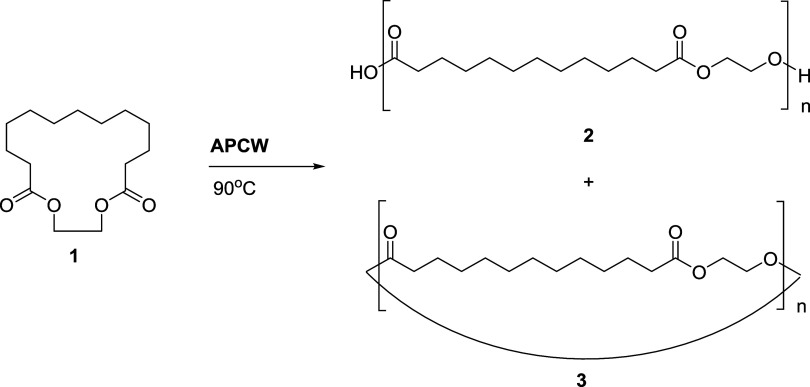
Screening of APCW-Catalyzed Polymerization^a^

entry	APCW (assembled components)	APCW (CALB, mg)^b^	time (h)	conv. (%)^c^	*n*^c^,^d^	*M*_w_ (g/mol)^e^
1	CALB	(3)	72	5	3	810
2	MCC	18	24			
3	H_2_O	20	24			
4	APCW1 (MCC/CALB/buffer)	30 (3.3)	5	80	8	2160
5	APCW2 (MCC/CALB/Brij/buffer)	30 (3)	2	94	8.5	2295
6	APCW3 (MCC/CALB)	30 (7.5)	4	80	18	4860
7	APCW4 (MCC/CALB/Brij)	10 (2)	3	80	15	4050
8	APCW4	30 (6)	1	93	11	2970
9	APCW5 (MCC/CALB/EB)	30 (6)	3	91	19	5130
10^f^	APCW5	30 (6)	8.5	90	25	6750
11^g^	tartaric acid	14	96	8		

a Ethylene brassylate **1** (300 mg, 1.11 mmol), **APCW** or **CALB**, 90 °C. The ratio of MCC/CALB/surfactant is 3:1:1.

b Amount of APCW used and amount of pure CALB component.

c Determined by ^1^H NMR.

d Number of monomeric units.

e Mw calculated from *M*_w_E.B_._* *n*.

f APCW5 was recovered from the entry 7 reaction and reused to catalyze the reaction.

g Reaction performed at 130 °C.

**Figure 2:**
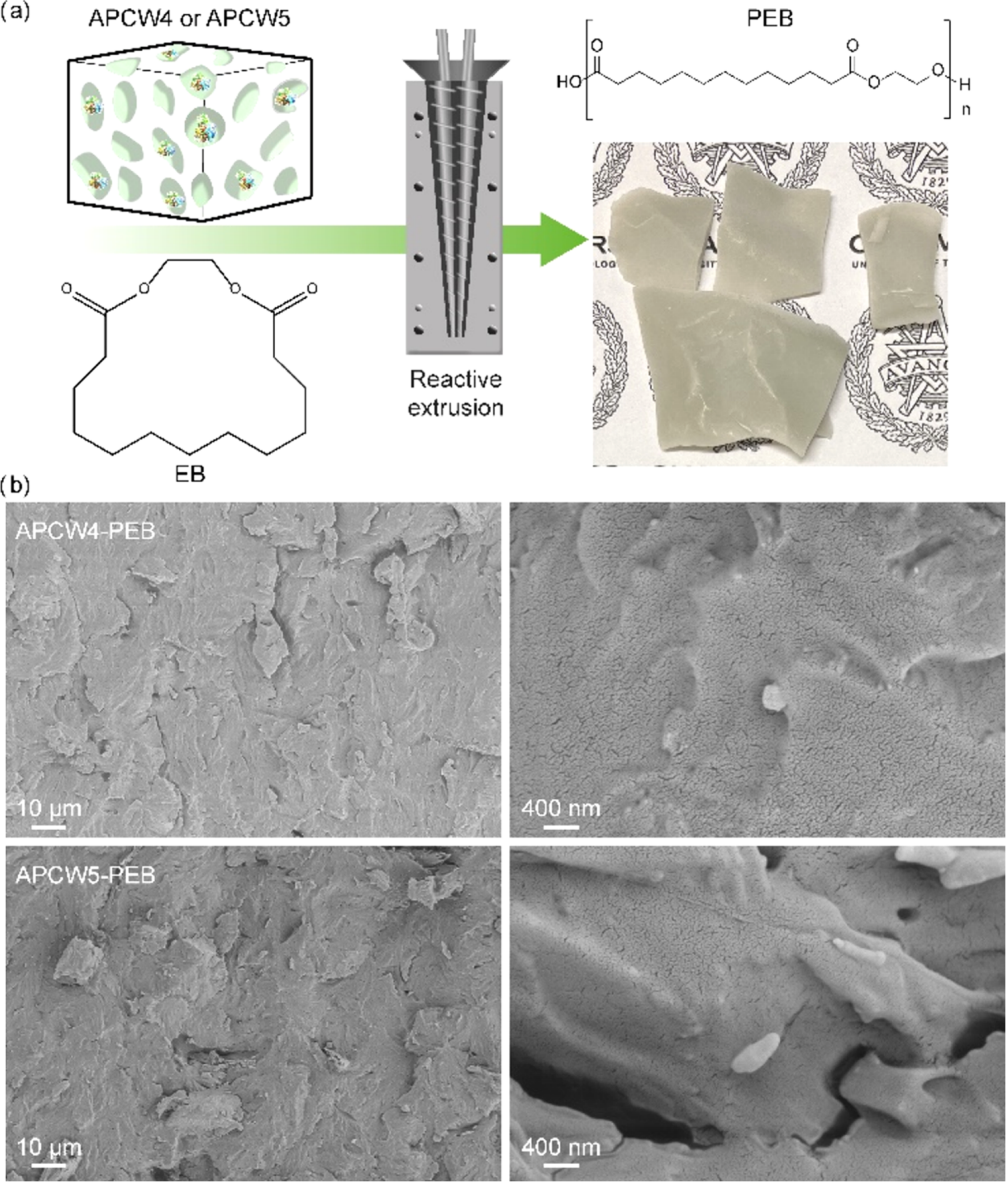
(a) Scheme of ring-opening polymerization of ethylene brassylate with APCW4 or APCW5 via reactive extrusion, with image of PEB. (b) Scanning electron microscopic images of cryo-fractured surfaces of PEB polymerized with APCW4 (top row) and APCW5 (bottom row) at two different magnifications.

The polymerization of ethylene brassylate catalyzed by APCW4 and APCW5 was also tested in reactive extrusion, a method that is potentially scalable and could provide faster kinetics for the polymerization compared to bulk and solvent methods. Both biocatalysts were utilized at a 10 wt % content, and at around 15 g of each material was produced during extrusion at 90 °C for up to 2 h. Macroscopically, both polymers were recovered as gray brittle solids (Figure 2a). After 30 min of reactive extrusion with the biocatalyst APCW4, the conversion of the monomer was 82% and the polymer reached a molar mass of 2000 g/mol measured by ^1^H NMR, which doubled after 2 h with a 98% conversion (entries 1 and 2, Table 2). In the same conditions, the polymerization catalyzed by APCW5 led to a lower molar mass (1400 g/mol) but the same conversion (98%) (entry 3, Table 2). The activity of APCW5 after the polymerization was also tested by reactive extrusion (entry 4, Table 2). The PEB obtained as product of the APCW5-catalyzed polymerization was mixed with a new monomer to verify the catalytic behavior of APCW5, without any intermediate purification, and/or whether the polymeric chains of PEB could be grown further. The obtained polymer had a similar average *M*_w_ (measured by SEC) to the PEB polymerized with pristine APCW5 and slightly higher polydispersity. As already observed in the bulk reaction, APCW5 activity was maintained even after reactive extrusion, enabling PEB polymerization to a similar extent of chain length.

**Table 2:** APCW-Catalyzed Polymerization in Reactive Extrusion

entry	APCW	REx time (min)	Mn_SEC_^a^ (g/mol)	*M*_w_SEC__^a^ (g/mol)	polydispersity^a^	conversion^b^ (%)	Mn^b^ (g/mol)
1	APCW4	30	4100	7600	1.8	82	2000
2	APCW4	120	6000	12 500	2.1	98	3900
3	APCW5	120	5300	10 900	2.0	98	1400
4^c^	APCW5	60	4400	11 100	2.5		

a Determined by SEC.

b Determined by ^1^H NMR.

c Reaction performed with 5 g of recycled PEB (entry 3) and 10 g of fresh EB.

**Figure 3:**
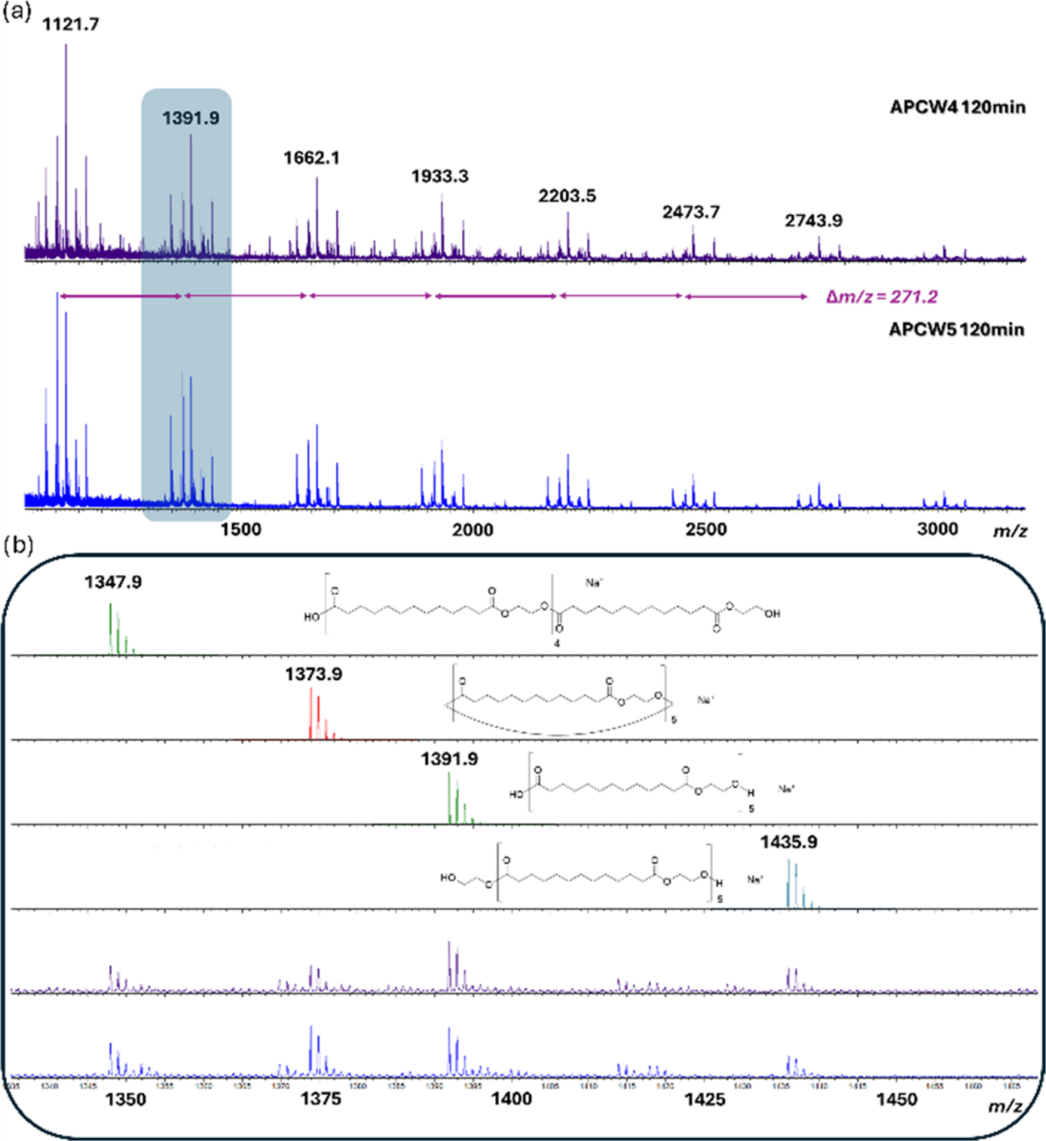
MALDI mass spectra for APCW4 and APCW5 after 120 min. (a) Extended view from 1000 to 3300 *m*/*z* and (b) magnification from 1300 to 1500. Comparisons with theoretical isotopic model are reported.

**Figure 4:**
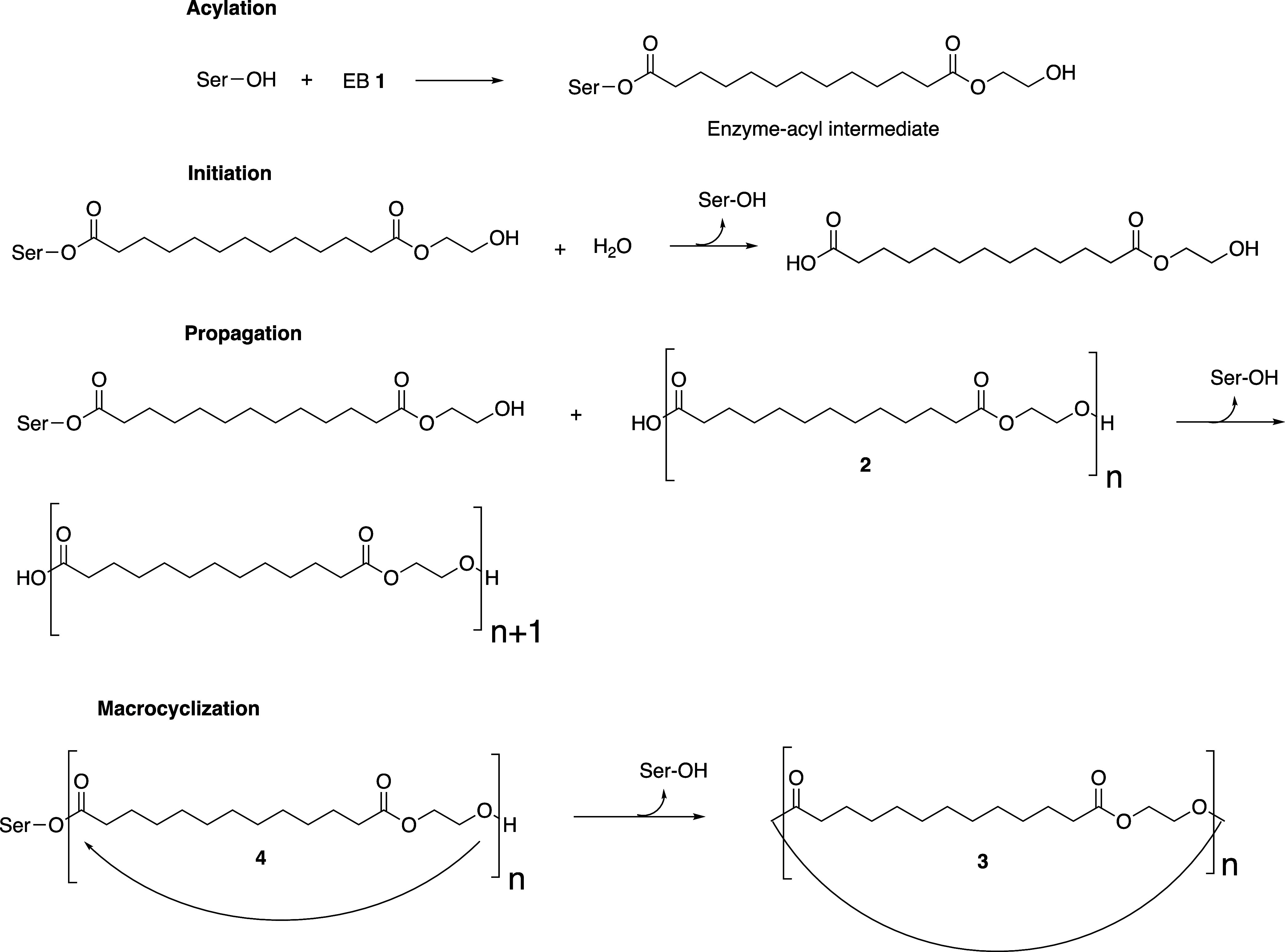
Simplified polymerization and macrocyclization mechanism of EB catalyzed by APCW. Ser = Serine105 of the CALB component of the APCW.

In order to acquire information about the structure of both polymers (entries 2 and 3, Table 2), matrix-assisted laser desorption/ionization (MALDI) mass analyses were performed. Polymer ions were detected from *m*/*z* 500 to at least *m*/*z* 10 000. Although no information about the mass parameters can be obtained as the molecular weight dispersity is higher than 1.2, the nature of the end-groups can be determined.^57^ As shown in Figure 3, a clear polymeric distribution is observed. The recurrent ion separation of 271.2u confirms the presence of ethylene brassylate oligomers. Interestingly, the polymer is quite clean since most of the end-groups are clearly identified and perfectly correspond to the predicted ones of **2** with carboxylic acid and hydroxyl end-groups as depicted in Figures 1 and 3. We also observed the presence of large ethylene brassylate macrocycles **3** (*n* = 2–7). The synthesis of macrocycles from simple lactones such as ε-caprolactone is an important feature of CALB catalytic activity.^58^ However, the ability of this enzyme to convert ethylene brassylate to macrocycles **3** has not been shown previously, to our knowledge. Thus, we found that APCW and CALB can catalyze the formation of macrocycles containing several ethylene brassylate units. The mechanism of the polymerization starts with acylation of the nucleophilic Ser105 within the active site of CALB by EB (Figure 4).^40,58^ This results in the formation of an enzyme-acyl intermediate. Next, the initiation step occurs via deacylation of the enzyme-acyl intermediate by a small amount of water to afford **2** (*n* = 1) and generate the nucleophilic Ser105 residue.^40,58^ This is determined by MALDI-TOF MS that determines the end-groups of **2** to have a carboxylic acid moiety. The propagation occurs by continued deacylation of the serine105-acyl intermediates by the hydroxyl end-groups of the growing polymer chain to afford the growing PEB **2**. In addition to forming acyl intermediates with EB, Ser105 also forms acyl intermediates with the produced PEB **2** to afford enzyme-acyl intermediates **4**.^58^ Intramolecular deacylation of acyl intermediates **4** affords the corresponding macrocycles **3**. The presence of **3** is determined by MALDI as described vide supra.

Thermogravimetric analysis (TGA) (Figure S4 and Table S2) was performed on the reactive extruded polymers, the monomer, and APCW4 and APCW5 to determine their thermal degradation behavior, which can provide further information on their macromolecular composition. The samples were characterized by two degradation steps, a minor one at around 340 °C and a major one at around 440 °C, related to the degradation of APCW and PEB, respectively. PEB polymerized by recycling APCW5 does not clearly show the first degradation step, possibly because of the lower amount of APCW compared to the other samples. The onset of thermal degradation, defined as the temperature at which 5% of weight loss occurs, increases with the reactive extrusion time. For PEB polymerized with APCW4, the onset increases from 295 to 318 °C, respectively, from 30 to 120 min, in agreement with the molecular weight changes, as longer polymer chains degrade at higher temperatures. PEB polymerized with APCW5 shows lower thermal stability at comparable processing times because of the slightly lower molecular weight and the lower thermal stability of APCW5 than APCW4.

The cryo-fractured surface of PEB (entries 2 and 3, Table 2) was analyzed by scanning electron microscopy to capture morphological features that can provide insights on the structure of these materials. Both samples have a brittle surface, in agreement with the low molecular weight of PEB (Figure 2b). Some voids are visible on the surfaces, together with microsized particles, ascribable to the MCC used as enzyme support. There is no visible debonding between the microparticles and the polymer matrix, indicating that the in situ polymerization of EB with APCW favors the adhesion between MCC and PEB.

**Figure 5:**
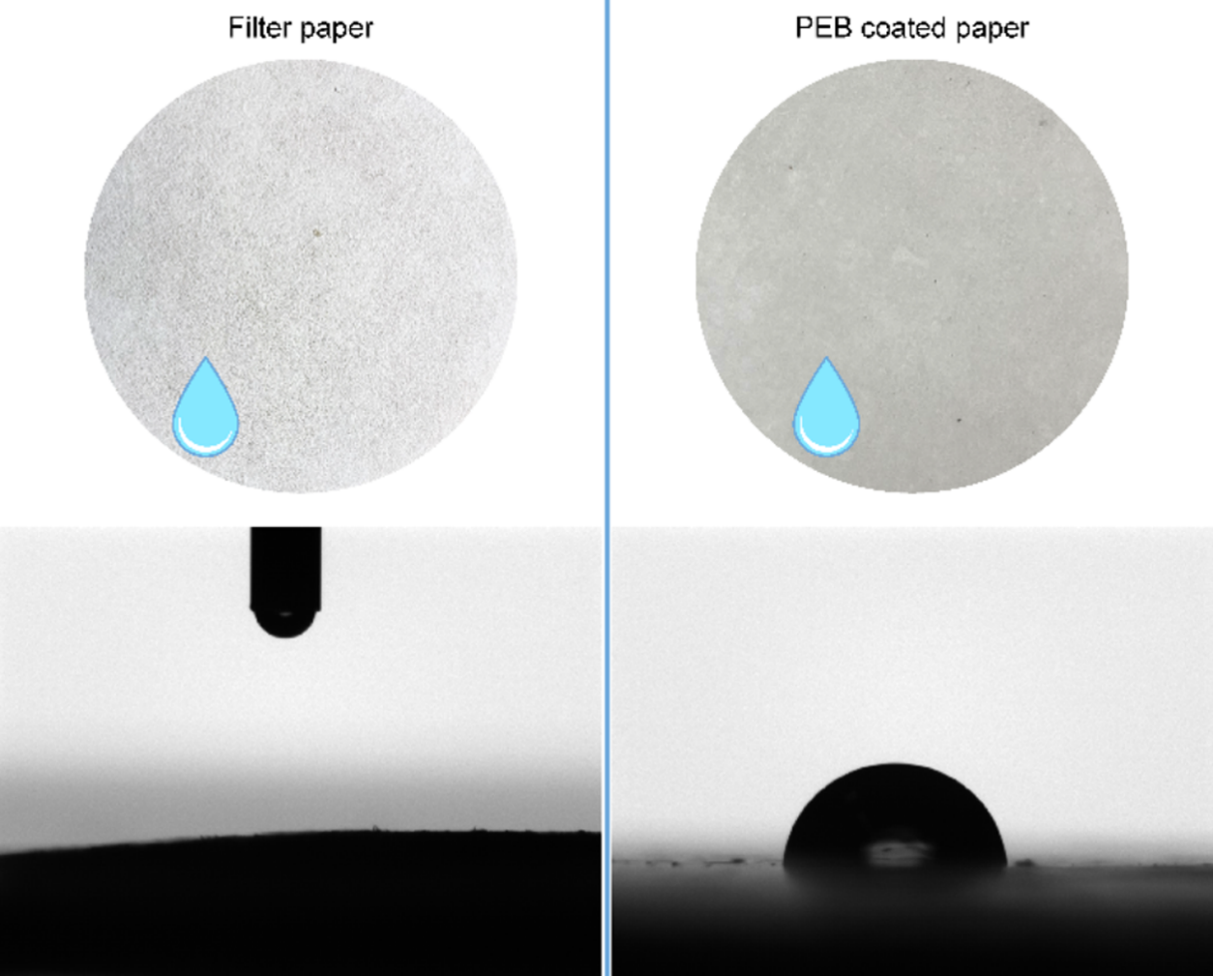
Photographs of pristine and PEB-coated filter paper with the respective photos of water contact angles after 15 s from drop deposition.

The application of PEB as a biobased hydrophobizing coating was tested using a filter paper as a substrate. The paper was coated by compression molding of PEB (entry 2, Table 2), and the water contact angle on the pristine and coated surfaces was measured. The pristine paper instantly absorbs water; therefore, its contact angle is 0°. Coating with PEB highly decreases the hydrophilicity of the surface, increasing the contact angle to 83 ± 4°, which is stable at least after 15 s (Figure 5).

## Conclusions

In this work, we disclosed that the concept of APCW assembly can improve the catalytic activity of enzymes for polymerization reactions in nonaqueous media. CALB-integrated APCWs were readily fabricated in an aqueous solution by self-assembly using MCC as the main component. The resulting APCW catalysts were tested in bulk reactions with the reactive extrusion technique leading, in both cases, to a high monomer conversion. The enzymatic stability of APCW was investigated by the reuse of the recycled catalyst in both bulk and reactive extrusion reactions. The application of PEB as a biobased and biodegradable hydrophobizing coating was tested on a filter paper showing an improvement in the hydrophobicity of the cellulosic material.

## Materials and Methods

### Materials

Avicel PH-101 (∼50 μm particle size), Lipase B *Candida antarctica* recombinant from *Aspergillus oryzae* (beige powder, ∼9 U/mg), Brij C10 (average Mn ∼ 683), and ethylene brassylate (1,4-dioxacycloheptadecane-5,17-dione) (>95%) were purchased from Sigma-Aldrich (Sweden). Before reaction, the monomer was dried in a ventilated oven overnight at 70 °C.

### General Procedure for the APCW Assembly with Brij as a Surfactant

In a plastic beaker were added MCC (60 mg), sodium phosphate buffer (6 mL, 0.1 M, pH = 7.2) or deionized H_2_O (6 mL), and Brij C10 (20 mg) (Table S1). The suspension was stirred with a spatula until complete solubilization of Brij C10. Next CALB (20 mg) was added, and the mixture was stirred with a spatula until complete solubilization of the enzyme and rapidly frozen in liquid nitrogen. The catalyst was lyophilized for 70 h to afford a solid white foam.

### General Procedure for the APCW Assembly with Ethylene Brassylate as a Surfactant

In a plastic beaker were added ethylene brassylate (20 mg), CALB (20 mg), and 1,4-dioxane (2 mL), and the solution was homogenized by stirring with a spatula (Table S1). The beaker was left uncovered, and dioxane was allowed to evaporate overnight under a fume hood. Next, deionized H_2_O (6 mL) and MCC (60 mg) were added, and the suspension was stirred with a spatula. The catalyst was lyophilized for 70 h to afford a solid white foam.

### Bulk Reactions

In an oven-dried microwave vial, ethylene brassylate (300 mg, 1.1 mmol) and APCW were added. The vial was capped and flushed with nitrogen. The reaction was stirred at 90 °C, and the conversion to poly(ethylene brassylate) was monitored by ^1^H NMR analysis taking a small aliquot of sample.

### Reactive Extrusion

Ethylene brassylate was polymerized via reactive extrusion in an Xplore microcompounder (15 cm^3^) at 90 °C and 100 rpm with a recirculating system for 120 min under a constant nitrogen flow. The polymerization was carried out with 10 wt % APCW4 or APCW5.

To evaluate the activity of the catalyst after polymerization, 5 g of PEB-APCW5 was extruded with 10 g of EB at 90 °C and 100 rpm for 60 min under a constant nitrogen flow.

### Paper Coating

A disk of cellulose filter paper (401, VWR) was coated with PEB (entry 2, Table 2) by compression molding with a manual press at 100 °C and a pressure of 12 ton for 2 min.

### Characterization Methods

^1^H NMR and ^13^C NMR experiments were carried out in solution-state conditions at 310 K on a Bruker AMX 500 MHz equipped with a 5 mm PABBO BB/19F-1H-D Z-GRD probe. CDCl_3_ was used as a solvent, and 0.3 wt % tetramethylsilane (TMS, 0 ppm) was used as an internal chemical shift reference. Spectra were recorded with a 12.0 ms pulse and 2 s relaxation delay. ^1^H–^1^H correlation spectroscopy (COSY) NMR experiment was performed with a 5.16 kHz spectral window using ^1^H 90° pulse width, F1 0.0249 s, F2 0.198 s acquisition time, 1 s relaxation delay, and 96 scans for 0.000194 increments.

SEC in chloroform was carried out at 30 °C using an Agilent (Diegem, Belgium) liquid chromatograph equipped with an Agilent degasser, an isocratic HPLC pump (flow rate = 1 mL min^–1^), an Agilent autosampler (loop volume = 100 μL; solution concentration = 2 mg mL^–1^), an Agilent-DRI refractive index detector, and three columns: a PL gel 5 μm guard column and two PL gel Mixed-B 5 μm columns (linear columns for separation of molecular weight (PS) ranging from 200 to 4 × 10^5^ g mol^–1^). Polystyrene standards were used for calibration.

Thermal stability was studied by thermogravimetric analysis with a TGA/DSC 3 + Star system (Mettler Toledo, Greifensee, Switzerland). Approximately 5 mg of each sample was preheated from room temperature to 70 °C, where an isothermal segment was maintained for 15 min to remove residual moisture. Then, the samples were heated to 500 °C at a heating rate of 5 °C min^–1^, under a nitrogen constant flow of 50 mL min^–1^.

Matrix-assisted laser desorption/ionization time-of-flight (MALDI-ToF) mass spectra were recorded using a Waters QToF Premier mass spectrometer. A Nd:YAG laser of 355 nm with a maximum pulse energy of 65 μJ delivered to the sample at a 50 Hz repeating rate was used. Time-of-flight mass analyses were performed in the reflection mode at a resolution of about 10 000. Trans-2-(3-(4-*tert*-butyl-phenyl)-2-methyl-2-propenylidene)malononitrile (DCTB) was used as the matrix and prepared as a 40 mg/mL solution in chloroform. The matrix solution (1 μL) was applied to a stainless steel target and air-dried. Polymer samples were dissolved in THF to obtain 1 mg/mL solutions, and 50 μL of NaI stock solution (2 mg mL^–1^ in acetonitrile) was added to the sample solution. 1 μL aliquots of these solutions were applied onto the target area (already bearing the matrix crystals) and air-dried.

PEB samples (entries 2 and 3, tale 2) were cryo-fractured in liquid nitrogen to analyze their morphology. The fractured surfaces were coated with gold for 1 min at 10 mA. The samples were then investigated using an Ultra 55 FEG scanning electron microscope (SEM) (Zeiss Sigma) under an accelerating voltage of 5 kV.

Water contact angle measurements were performed on the PEB-coated paper using an Attension Theta optical tensiometer, by Biolin Scientific. The mean contact angle of Milli-Q water was measured for 15 s since the drop deposition on the coated paper. An average of six measurements was considered. A cellulose filter paper was used as a reference.

## Supporting Information Available

General procedures for APCW assembly; ^1^H NMR, ^13^C NMR, and ^1^H–^1^H COSY NMR of PEB, TGA of PEB and reagents (PDF)
