# Supplementary material for: In Situ Enzymatic Polymerization of Ethylene Brassylate Mediated by Artificial Plant Cell Walls in Reactive Extrusion
Source: ACS Appl Polym Mater. 2024 Aug 16;6(17):10414–22. doi: 10.1021/acsapm.4c01568 (PMC11406489; doi:10.1021/acsapm.4c01568)
Supplement: Supplementary file 1 — ap4c01568_si_001.pdf [file ap4c01568_si_001.pdf]

Supplementary Information

In-situ enzymatic polymerization of ethylene brassylate mediated by artificial plant cell walls in reactive extrusion

Luca Deiana,[a] Angelica Avella,[b] Abdolrahim A. Rafi,[a] Rosica Mincheva,[c] Julien De Winter,[d] Giada Lo Re,*[b] Armando Córdova*[a]

[a] Department of Natural Sciences, Mid Sweden University, Holmgatan 10, Sundsvall, 85179, Sweden, E-mail: armando.cordova@miun.se

[b] Department of Industrial and Materials Science, Chalmers University of Technology, Rännvägen 2a, Gothenburg, 41258, Sweden, E-mail: giadal@chalmers.se

[c] Laboratory of Polymeric and Composite Materials, University of Mons (UMONS), 7000 Mons, Belgium

[d] Organic Synthesis and Mass Spectrometry Laboratory (S2MOs), University of Mons (UMONS), 7000 Mons, Belgium

Table of contents

General methods…………………………………………………………..S2

Experimental procedure…………………………………………………S3

General methods:

Chemicals and solvents were either purchased from commercial suppliers or purified by standards techniques. Avicel® PH-101 (~50 μm particle size), Lipase B Candida antarctica recombinant from Aspergillus oryzae (beige powder, ~9 U/mg), Brij® C10 (average Mn ~683), ethylene brassylate were purchased from Aldrich and used as received. Dry toluene was column-dried directly before use by a VAC: Solvent Purifier system. ^{1}H NMR spectra were recorded on a Bruker Avance 500 (500 MHz) spectrometer. Chemical shifts are reported in ppm from tetramethylsilane with the solvent resonance resulting from incomplete deuterium incorporation as the internal standard (CDCl3: δ 7.26 ppm). Data are reported as follows: chemical shift, multiplicity (s = singlet, d = doublet, q = quartet, br = broad, m = multiplet), and coupling constants (Hz), integration. Chemical shifts are reported in ppm from tetramethylsilane with the solvent resonance as the internal standard (CDCl3: δ 77.26 ppm). Unless otherwise noted, all reactions were performed with distilled solvents in oven- dried (160°C) glassware. and chemical consultancy services (United Kingdom) by ICP-OES. Infrared spectra were recorded by Thermo Scientific NICOLET 6700 FT-IR, Smart orbit, Diamond 30000-200 cm^{-1}.

General procedure for the assembling of artificial plant cell wall (APCW) with Brij as surfactant.

In a plastic beaker was added MCC (60 mg), sodium phosphate buffer (6 mL, 0.1 M, pH 7.2) or deionized H_2O (6 mL) and Brij C10 (20 mg). The suspension was stirred with a spatula until completely solubilization of Brij C10. Next CALB (20 mg) was added, the mixture was stirred with a spatula until completely solubilization of the enzyme and rapidly frozen in liquid nitrogen. The catalyst was lyophilized for 70 hours to give a solid white foam. APCW was stored in a desiccator containing a saturated LiCl solution to obtain the water activity (aw) of 0.5.

General procedure for the assembling of artificial plant cell wall (APCW) with ethylene brassylate as surfactant.

In a plastic beaker was added ethylene brassylate (20 mg), CALB (20 mg) and 1,4-dioxane (2 mL) and the solution was homogenized stirring with a spatula. The beaker was left uncovered and dioxane was allowed to evaporate overnight under a fume hood. Next, deionized H_2O (6 mL) and MCC (60 mg) were added and the suspension was stirred with a spatula. The catalyst was lyophilized for 70 hours to give a solid white foam. APCW was stored in a desiccator containing a saturated LiCl solution to obtain the water activity (aw) of 0.5.

Table S1. Reagents for the assembling of APCW.

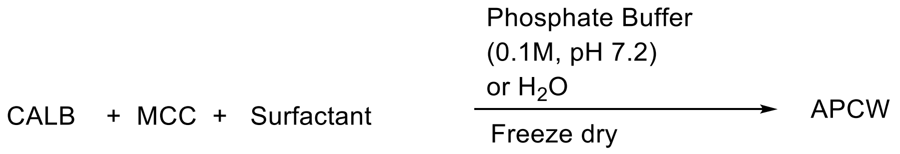

Catalyst	CALB (mg)	MCC (mg)	Surfactant (mg)
**APCW1** (MCC/CALB/buffer)[a]	20	60	-
**APCW2 (**MCC/CALB/Brij/buffer)[a]	20	60	20
**APCW3 (**MCC/CALB/H_2O)[b]	20	60	-
**APCW4 (**MCC/CALB/Brij/H_2O)[b]	20	60	20
**APCW5 (**MCC/CALB/EB/ H_2O)[b]	20	60	20

[a] Sodium phosphate buffer (6 mL, 0.1 M, pH 7.2) used as solvent. [b] Deionized H_2O (6 mL) used as solvent.

General procedure for the APCW catalyzed polymerization of ethylene brassylate.

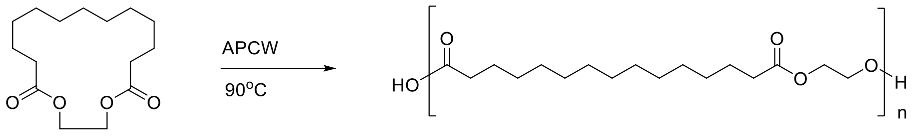

In a oven dried microwave vial were added ethylene brassylate (300 mg, 1.1 mmol) and APCW. The vial was capped and flushed with nitrogen. The reaction was stirred at 90°C and the conversion to poly ethylene brassylate was monitored by ^{1}H-NMR analysis taking small aliquot of sample.

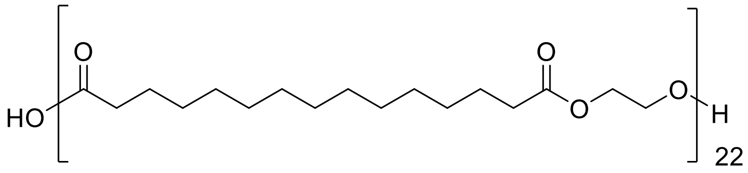

^{1}H NMR (500 MHz, $CDCl_3$) δ 4.24 (s, 89H), 4.20 – 4.18 (m, 2H), 3.83 – 3.77 (m, 2H), 2.29 (t, J = 7.5 Hz, 96H), 1.68 – 1.49 (m, 96H), 1.25 (d, J = 12.6 Hz, 346H). ^{13}C NMR (126 MHz, $CDCl_3$) δ 174.2, 173.7, 69.2, 66.0, 63.3, 62.1, 61.3, 34.2, 29.6, 29.5, 29.3, 29.2, 24.9.

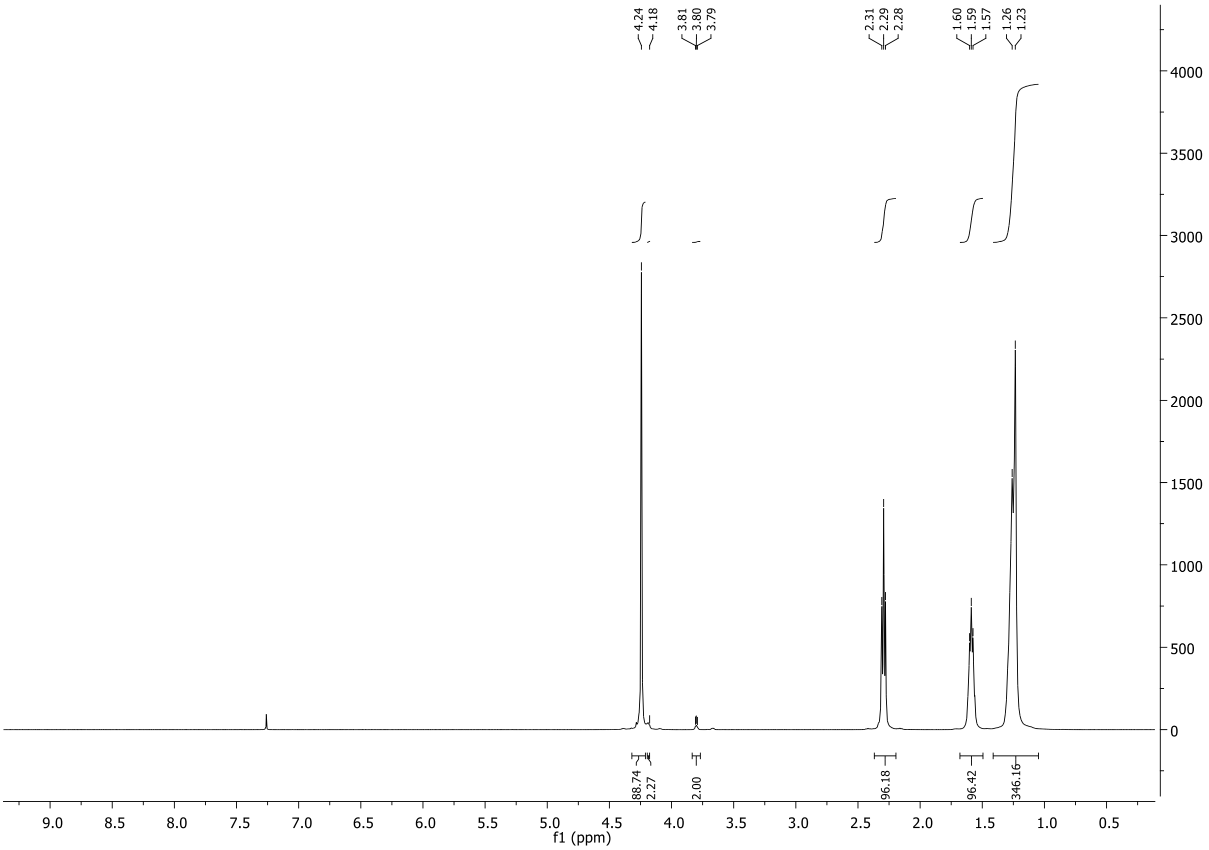

Figure S1. ^{1}H-NMR spectrum of PEB in $CDCl_3$.

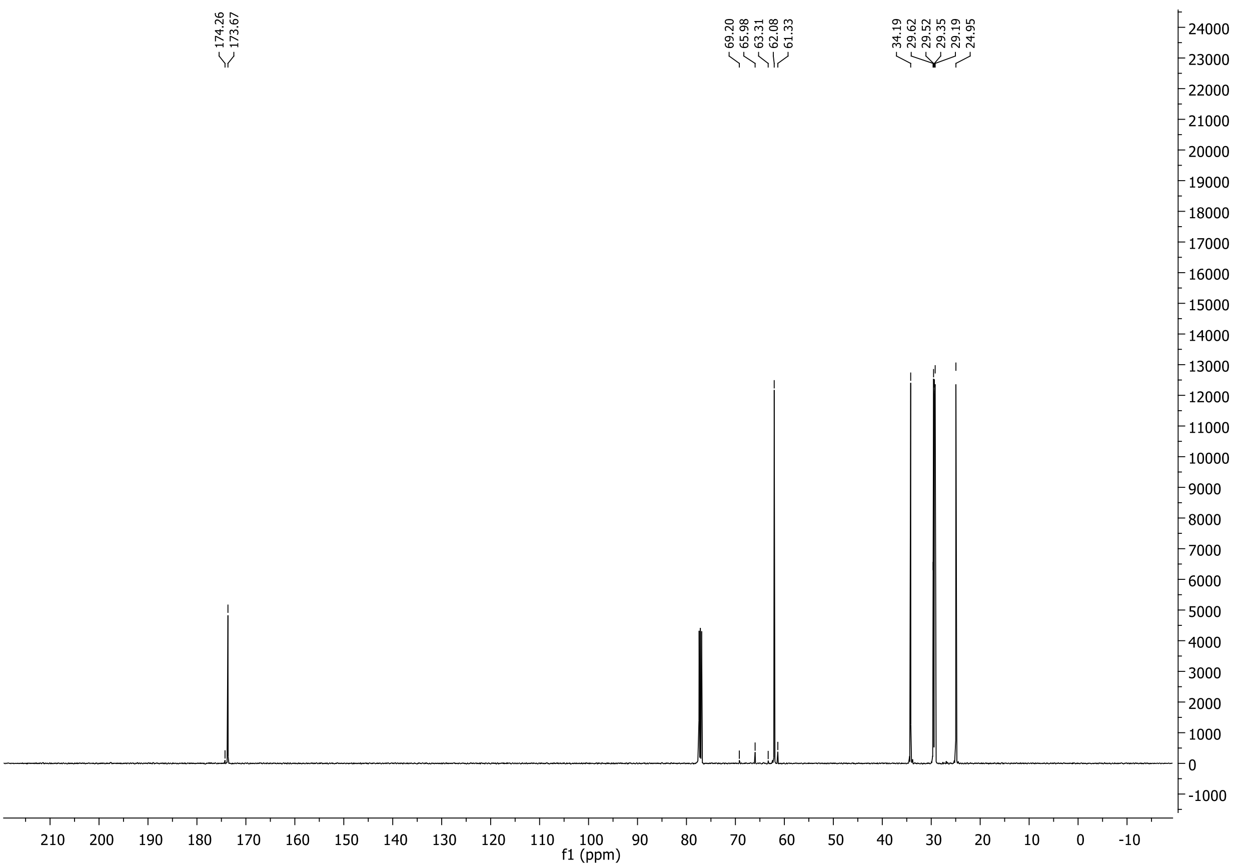

Figure S2. ^{13}C-NMR spectrum of PEB in $CDCl_3$.

^1H-^1H Correlation spectroscopy nuclear magnetic resonance (COSY NMR)

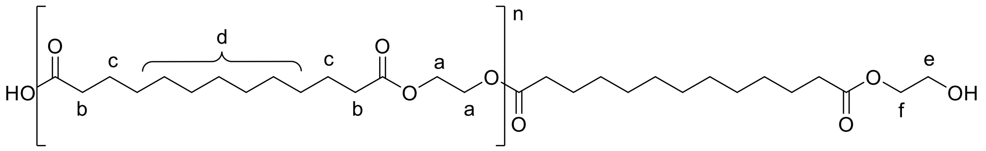

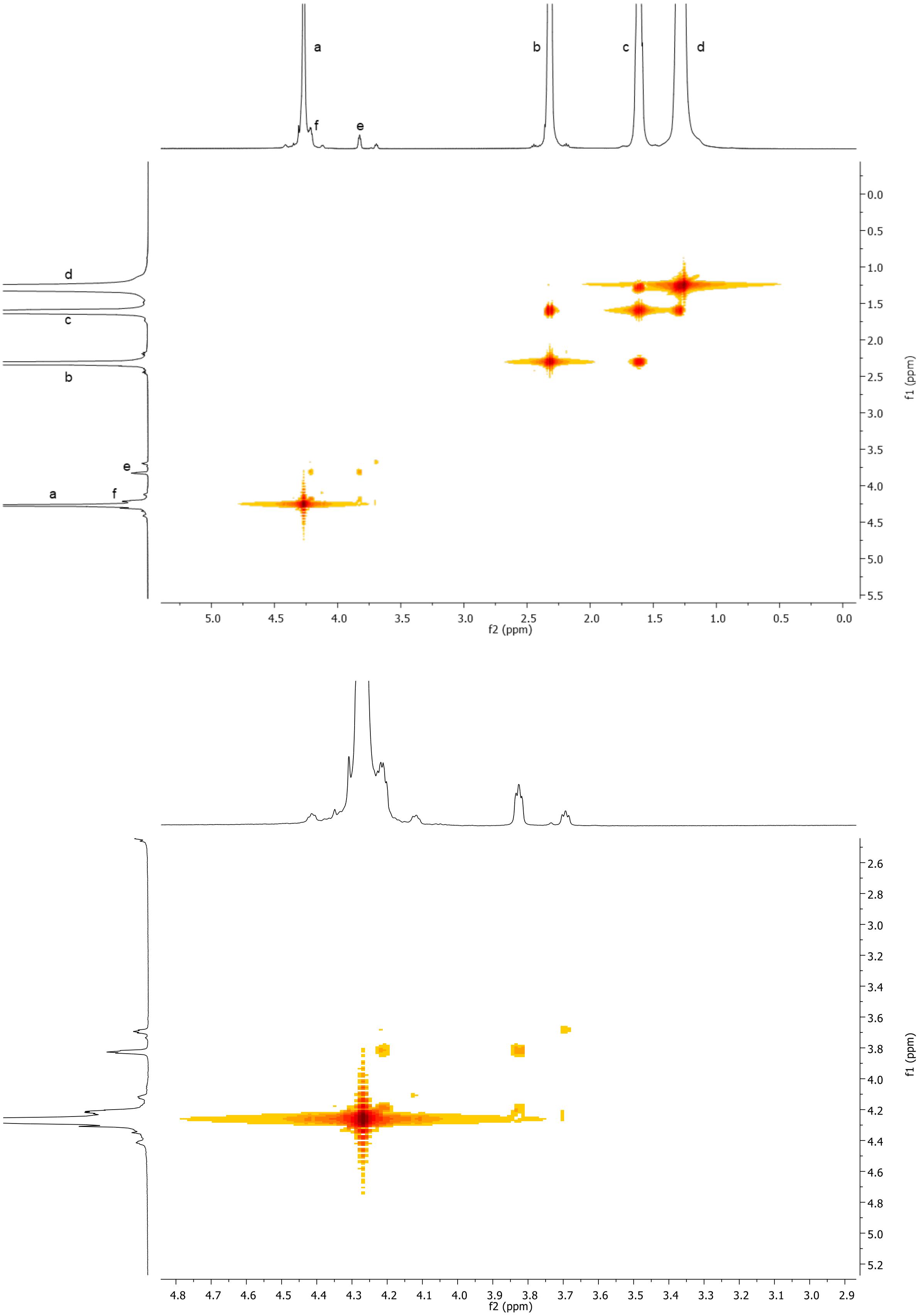

Figure S3. ^{1}H-^{1}H COSY NMR of poly(ethylene brassylate) with H_2O as initiator.

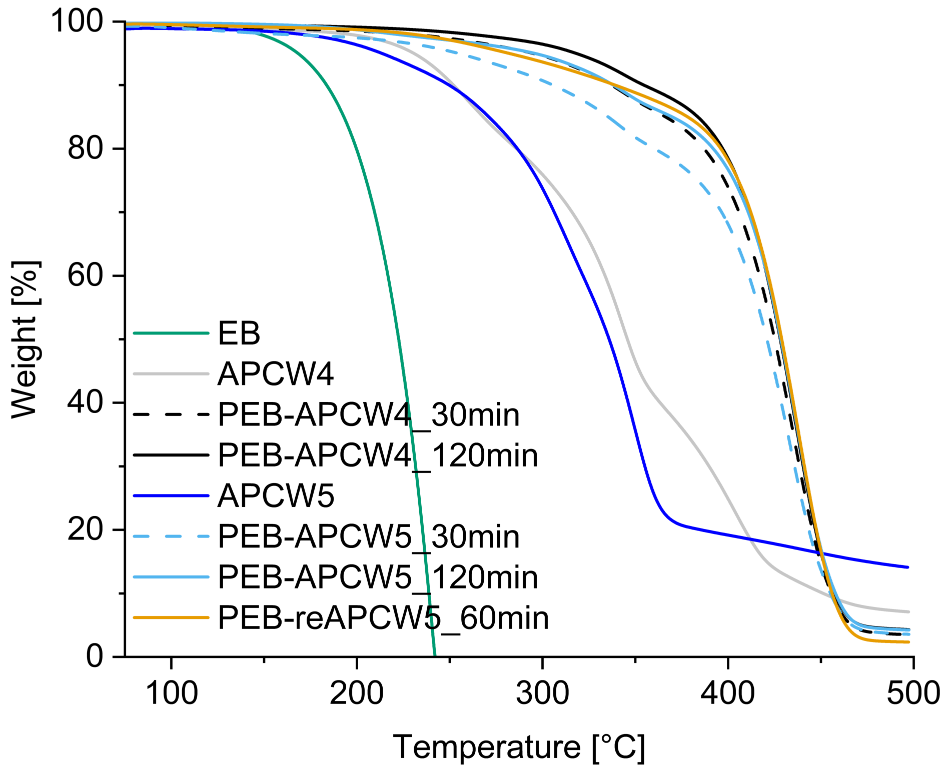

Figure S4. Thermogravimetric analysis in N_2 of PEB, EB, APCW4 and APCW5.

Table S2. Onset of thermal degradation temperatures ($T_{5\%}$), degradation temperatures (T_d) and char residues at 500 °C measured by thermogravimetric analysis in N_2.

**Sample**	$T_{5\%}$ **[°C]**	T_d **[°C]**	**Char residue [%]**
EB	168	240	0
APCW4	231	261, 344, 405	7
PEB-APCW4_30min	295	342, 436	3
PEB-APCW4_120min	318	344, 438	4
APCW5	214	225, 312, 350	14
PEB-APCW5_30min	255	342, 435	4
PEB-APCW5_120min	296	346, 438	4
PEB-reAPCW5_60min	282	438	2
